# D-dimer/high sensitive troponin I ratio is useful in predicting in-hospital mortality in pulmonary embolism patients

**DOI:** 10.1007/s11845-024-03766-7

**Published:** 2024-07-30

**Authors:** Ahmet Burak Urfalioglu, Ertugrul Altug, Hayri Cinar, Erdem Aksay, Onder Yesiloglu, Adem Cakir, Mustafa Avsar, Ozlem Ercen Diken, Ramazan Guven, Akkan Avci

**Affiliations:** 1 Department of Emergency Medicine, Health Science University, Adana City Research and Training Hospital, Kışla Mahallesi, Dr. Mithat Özsan Bulvarı, 4522. Sokak No: 28, Yüreğir/Adana, Turkey; 2 Department of Emergency Medicine, Health Science University, Istanbul Cam and Sakura City Research and Training Hospital, Istanbul, Turkey; 3 Department of Emergency Medicine, Health Science University, 25 Aralik State Hospital, Gaziantep, Turkey; 4 Canakkale Mehmet Akif Ersoy State Hospital, Canakkale, Turkey; 5 Emergency Medicine Clinic, Yuregir State Hospital, Adana, Turkey; 6 Department of Thoracic Diseases, Health Science University, Adana City Reseach and Training Hospital, Adana, Turkey

**Keywords:** D-dimer, Emergency, HsTroponin, Mortality, Pulmonary embolism

## Abstract

**Background:**

Pulmonary embolism requires careful differential diagnosis as it is associated with a wide range of symptoms that may suggest different diseases such as chest pain, shortness of breath and syncope. Since the disease can be fatal, especially in cases where right ventricular failure and hemodynamic instability develop, prognostic markers are great importance in terms of monitoring the patient during the treatment process.

**Aim:**

We aimed in our study to compare the relationship between the ratio of D-dimer and High Sensitive Troponin T (HsTnT) values ​​with short-term mortality and to compare this relationship with Pulmonary Embolism Severity Index (PESI) scoring.

**Method:**

Our study was conducted with patients who applied to the emergency department of our hospital between 01/01/2022 and 01/01/2023 and were definitively diagnosed with Pulmonary thromboembolism after their evaluation.

Findings.

The success of D-dimer/HsTroponin, D-dimer/CK-MB and troponin/D-dimer indices calculated from the laboratory test results of the cases in predicting mortality was examined, and a comparison was made with the success of the PESI score in predicting mortality. Among these indices, D-dimer/CK-MB was found to be the most successful index in predicting 7-day mortality (AUC: 0.734; 95% CI: 0.653–0.815; *p* < 0.001). Additionally, the D-dimer/HsTroponin ratio was found to be statistically significant as a successful index in predicting 7-day mortality (AUC: 0.697; 95% CI: 0.621–0.774; *p* < 0.001).

**Conclusion:**

FD-dimer/HsTroponin ratio, which is a powerful, fast, low-cost, easy and simple test, can be used especially in emergency services instead of the PESI score as a mortality marker in pulmonary embolism, which has a high mortality rate.

## Introduction

Acute pulmonary embolism (PE) is among the cardiovascular emergencies whose diagnosis and treatment process usually begins in the emergency room and can be fatal if not managed appropriately. PE, which is a thromboembolic complication with an annual incidence of 39–115 per 100,000 applications, requires careful differential diagnosis as it is associated with a wide range of symptoms that may suggest different diseases such as chest pain, shortness of breath and syncope [1]. Since the disease can be fatal, especially in cases where right ventricular failure and hemodynamic instability develop, prognostic markers are great importance in terms of monitoring the patient during the treatment process [2]. Today, scores such as Pulmonary Embolism Severity Index (PESI), which evaluates gender, history of chronic diseases (cancer, heart failure, and chronic lung disease), heart rate, systolic blood pressure, respiratory rate, body temperature, altered mental status and oxygen saturation, are used to determine the risk status in pulmonary embolism patients; alternative laboratory methods such as H-FABP analysis and prognostic methods for low-risk patient groups such as BOVA scoring also appear as prognostic predictive alternatives [3, 4]. However, it is not always possible to apply these methods effectively in emergency room conditions due to reasons such as density and limited resources.

D-dimer is a strong diagnostic excluder in pulmonary embolism, and its level has been reported to be associated with pulmonary obstruction and right ventricular failure, and therefore the severity of the disease [5], but its long-term mortality predictive effect is not thought to be strong [6]. There is a general consensus that troponin values, another cardiovascular laboratory parameter, are successful in assessing the 30-day mortality risk and predicting outcomes in patients with pulmonary embolism; however, this effectiveness varies between studies [7]. The fact that D-dimer is a protein product resulting from fibrin degradation and that it increases in many cases where the coagulation cascade is activated, and that troponin values ​​and especially the high-sensitive subgroup are sensitive to cardiac diseases related to myocardiocyte damage, limits the prognostic value of these two markers alone in pulmonary embolism.

Recently, studies have been conducted showing the effectiveness of using D-dimer and different Troponin species values ​​together in the differential diagnosis of cardiovascular diseases such as myocardial infarction, aortic aneurysm and pulmonary embolism [8–10], but, there is insufficient data in the literature regarding the evaluation of these two biomarkers together as a prognostic parameter predicting mortality. In order to shed light on this issue, we aimed in our study to compare the relationship between the ratio of D-dimer and High Sensitive Troponin T (HsTnT) values ​​with short-term mortality and to compare this relationship with PESI scoring.

## Methods

### Study design

Our study was planned retrospectively at Adana City Training and Research Hospital. Our study was conducted between 01/12/2023 and 01/03/2024. Before starting the study, ethics committee approval (Ethics committee decision number: 2731, Date: 08/03/23) was obtained from the ethics committee.

**Fig. 1:**
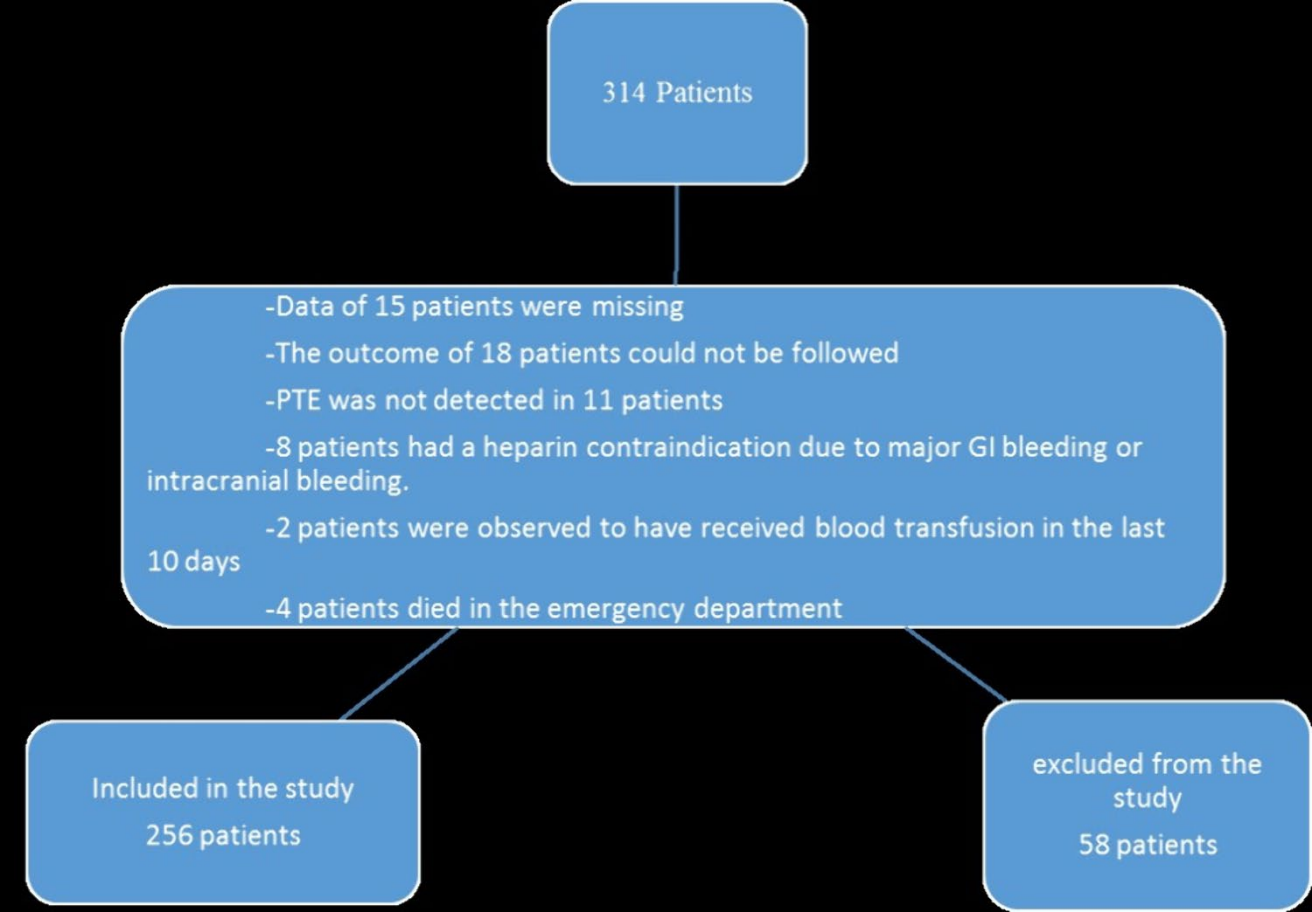
Flow chart

Our study was conducted with patients who applied to the emergency department of our hospital between 01/01/2022 and 01/01/2023 and were definitively diagnosed with Pulmonary thromboembolism (PTE) after their evaluation. The cases were identified by scanning the hospital automation system and patient files. While scanning in the automation system, ICD 10 diagnosis codes “I26., I26.0 and I26.9” were used for PTE. Patients meeting exclusion criteria were excluded from the 314 patients obtained as a result of this screening (Fig. 1).

**Fig. 2:**
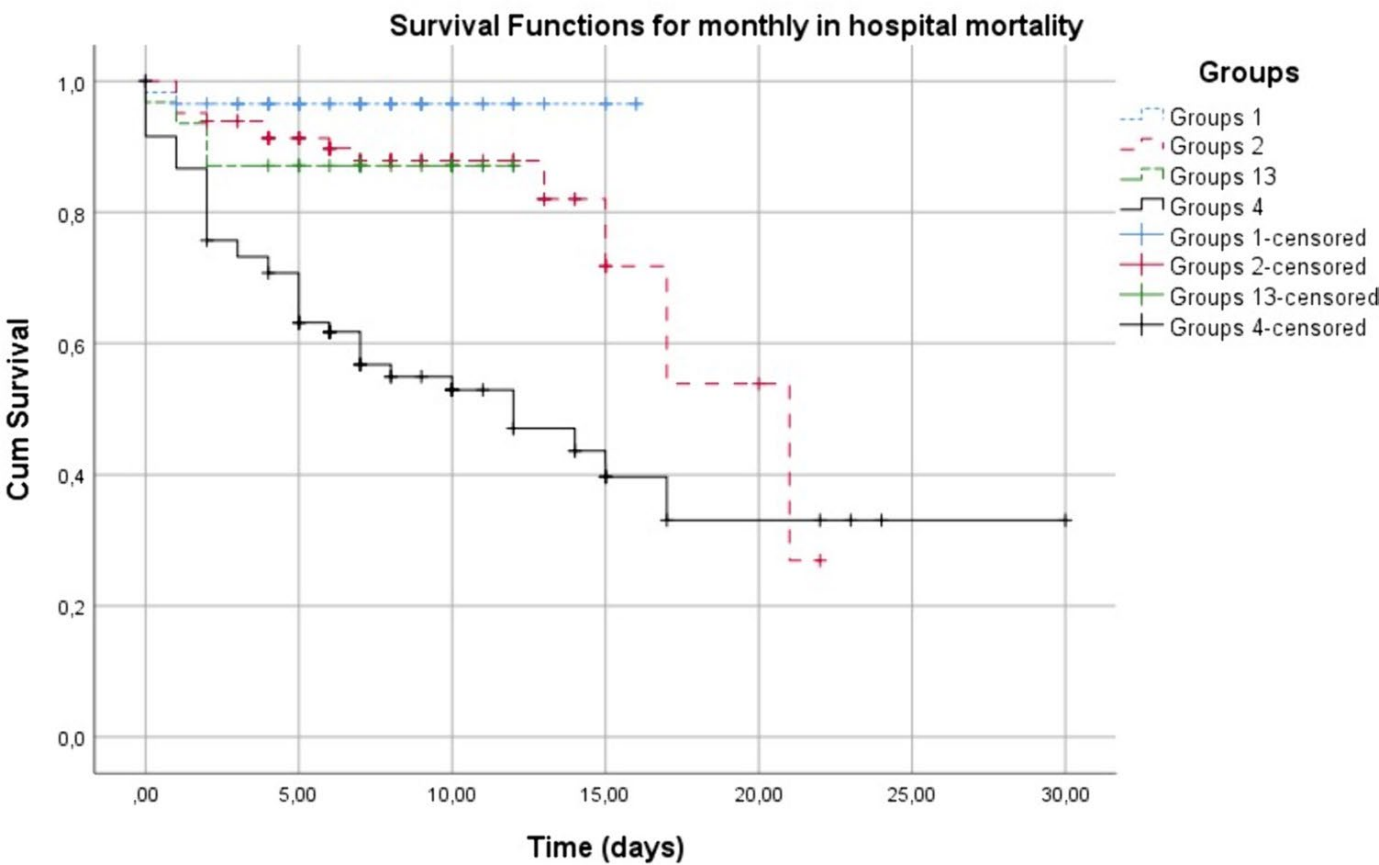
Survey evaluation of groups formed according to sPESI, troponin and CK-MB positivity according to 7-day, 14-day and 30-day periods

Demographic data, vital data and clinical data of the cases were obtained from the hospital automation system, patient files and ward admission files. The cases were divided into four groups according to cardiac troponin (high sensitivity troponin) and sPESI scoring. Group 1: troponin negative and low risk (LR) sPESI; Group 2: troponin positive and high risk (HR) sPESI; Group 3: troponin negative and LR sPESI and Group 4: troponin positive and HR sPESI cases (Fig. 2).

### Patient monitoring

Low and medium risk patients followed in our hospital were given dose-adjusted standard heparin (80 IU/kg bolus and 18 IU/kg infusion) or low molecular weight Heparin (enoxaparin 2 × 100 IU/kg/day). In high-risk and selected intermediate-risk PTE patients, 100 mg/2 h IV-tPA (tissue plasminogen activator) followed by standard heparin treatment was applied.

### Diagnostic evaluation

In the study, the high sensitive troponin-I values ​​of the patients measured at admission were determined using a quantitative electrochemiluminescence immunoassays test (Elecsys 2010; Roche, Mannheim, Germany). A positive troponin test result and a troponin level above the manufacturer’s test threshold were accepted for the diagnosis of myocardial damage.

### Statistical analysis

Data were analyzed with SPSS Package Program version 26.0. Number, percentage, mean, standard deviation, median, minimum and maximum were used to present descriptive data. The suitability of the data for normal distribution was evaluated with the Kolmogorov–Smirnov test. In univariate analysis, continuous variables with normal distribution were expressed as mean ± SD and compared using the *t* test. Pearson Chi-square test was used to analyze categorical variables. For categorical variables, Fisher’s exact test was used if there were fewer than five variables. *t* test was used for comparison of two independent numerical data. Kaplan–Meier analysis was used to determine cause-related surveys. To determine diagnostic accuracy and prediction success, it was evaluated using ROC (receiver operating characteristic) curve analysis. Appropriate cut-off values ​​were determined, and sensitivity and specificity values ​​were calculated for parameters with area under the curve (AUC) above 0.600.

*p* < 0.05 was accepted as the level of statistical significance.

## Results

Of the 256 cases included in our study, 46.5% were male, and the average age was 67.62 ± 15.42 years. When the average vital parameters of the cases were examined, it was observed that there was an elevated pulse and low saturation. It was determined that sinus tachycardia most frequently developed in the cases (52.0%) and mortality was observed in 23.4% (*n* = 60) of the cases. When the cases are evaluated according to their outcomes, it was observed that mortality was higher in female cases (*p* = 0.042) and the average age was significantly higher (*p* < 0.001) in mortal cases. In cases with mortality, systolic BP, diastolic BP, MAP and saturation levels were significantly lower; The average pulse rate was found to be significantly higher. No significant relationship was found between fever and outcome, while D-dimer elevation was significantly higher (*p* < 0.001) in cases with mortality. It was observed that sinus tachycardia and AF were significantly higher in cases with mortality than in living cases. Again, in cases with exitus, it was observed that the sPESI level (HR) was high (*p* < 0.001), troponin positivity was high (*p* < 0.001) and the length of stay was significantly low (*p* < 0.001). In these cases, D-dimer/troponin and D-dimer/CK-MB, calculated by laboratory tests troponin, CK-MB and D-dimer levels, were significantly higher (*p* < 0.001 and *p* = 0.021, respectively); troponin/D-dimer level was found to be significantly low (*p* = 0.010) (Table 1).

**Table 1:** Comparison of demographic and clinical data of the cases according to total and in-hospital mortality

Parameter	Total (*n* = 256) *n* (%)/mean ± SD	In-hospital mortality		*p*
		Exitus (*n* = 60) *n* (%)/mean ± SD	Alive (*n* = 196) *n* (%)/mean ± SD	
Age (years)	67.62 ± 15.42	75.40 ± 13.89	65.23 ± 15.11	** < 0.001**
Gender (male)	119 (46.5)	21 (35.0)	98 (50.0)	0.042
Vital parameters				
Systolic blood pressure (mm-Hg)	119.32 ± 26.92	111.58 ± 27.64	121.68 ± 26.31	**0.011**
Diastolic blood pressure (mm-Hg)	74.83 ± 15.09	69.72 ± 17.76	76.40 ± 13.85	**0.003**
Mean arterial pressure (mm-Hg)	89.68 ± 17.30	84.63 ± 20.71	91.23 ± 15.85	**0.009**
Pulse (beat/min)	106.68 ± 20.94	116.25 ± 22.86	103.74 ± 19.46	** < 0.001**
Saturation (%)	90.03 ± 8.15	84.63 ± 20.71	91.23 ± 15.85	** < 0.001**
Fever (°C)	36.65 ± 0.52	36.72 ± 0.50	36.63 ± 0.52	0.210
Laboratory test				
hsTnI (ng/L)	171.51 ± 432.09	80.05 ± 75.16	199.50 ± 488.96	0.061
CK-MB (µg/L)	4.33 ± 8.91	4.85 ± 7.12	4.17 ± 9.41	0.604
D-dimer (mcg/L)	12,818.34 ± 15,372.32	27,349.37 ± 24,222.48	8370.07 ± 6829.16	** < 0.001**
hsTnI status				
Negative	90 (35.2)	6 (10.0)	84 (42.9)	** < 0.001**
Positive	166 (64.8)	54 (90.0)	112 (57.1)	
Electrocardiography results				
Normal sinus rhythm	98 (38.3)	13 (21.7)	85 (43.4)	**0.004**
Sinus tachycardia	133 (52.0)	37 (61.7)	96 (49.0)	
Atrial fibrillation	25 (9.8)	10 (16.7)	15 (7.7)	
PESI score	107.44 ± 35.52	118.02 ± 29.93	104.20 ± 36.52	**0.008**
sPESI				
sPESI < 1 (low risk)	142 (55.5)	15 (25.0)	127 (64.8)	** < 0.001**
sPESI ≥ 1 (high risk)	114 (44.5)	45 (75.0)	69 (35.2)	
Emergency follow-up times (h)	465.48 ± 550.59	484.60 ± 463.23	4558.71 ± 575.59	0.723
Hospitalization stays (days)	7.57 ± 4.91	4.67 ± 5.10	8.46 ± 4.50	** < 0.001**
Indexes				
D-dimer/hsTnI	578.68 ± 1214.56	893.95 ± 1958.20	482.17 ± 853.76	**0.021**
D-dimer/CK-MB	5984.21 ± 8880.84	11,417.39 ± 13,875.49	4320.99 ± 5746.39	** < 0.001**
hsTnI/D-dimer	0.02 ± 0.06	0.007 ± 0.009	0.029 ± 0.066	**0.010**

*hsTnI* high sensitive troponin-I, *CK-MB* creatine kinase-MB, *PESI* Pulmonary Embolism Severity Index

Values of *p*<0.05 are indicated in bold

The 7-day, 14-day and 30-day in-hospital mortality levels of the cases were compared according to groups, and it was seen that the mortality level increased significantly as troponin positivity and sPESI risk increased at both 7-day, 14-day and total in-hospital mortality levels (*p* < 0.001 for all comparisons) (Table 2).

**Table 2:** Examination of the relationship between the 7-day, 14-day and 30-day mortality levels of the groups formed according to the sPESI and troponin positivity of the cases

**Groups**	**Parameters**	**In-hospital mortality (7 days)**		***p***
		**Alive (*n* = 207)** ***n* (%)**	**Exitus (*n* = 49)** ***n* (%)**	
**1**	Low risk and hsTnI −	57 (96.6)	2 (3.4)	** < 0.001**
**2**	Low risk and hsTnI +	74 (89.2)	9 (10.8)	
**3**	High risk and hsTnI −	27 (87.1)	4 (12.9)	
**4**	High risk and hsTnI +	49 (59.0)	34 (41.0)	
**Groups**	**Parameters**	**In-hospital mortality (14 days)**		***p***
		**Alive (*n* = 201)** ***n* (%)**	**Exitus (*n* = 55)** ***n* (%)**	
**1**	Low risk and hsTnI −	57 (96.6)	2 (3.4)	** < 0.001**
**2**	Low risk and hsTnI +	73 (88.0)	10 (12.0)	
**3**	High risk and hsTnI −	27 (87.1)	4 (12.9)	
**4**	High risk and hsTnI +	44 (53.0)	39 (47.0)	
**Groups**	**Parameters**	**In-hospital mortality (30 days)**		***p***
		**Alive (*n* = 207)** ***n* (%)**	**Exitus (*n* = 49)** ***n* (%)**	
**1**	Low risk and hsTnI −	57 (96.6)	2 (3.4)	** < 0.001**
**2**	Low risk and hsTnI +	70 (84.3)	13 (15.7)	
**3**	High risk and hsTnI −	27 (87.1)	4 (12.9)	
**4**	High risk and hsTnI +	42 (50.6)	41 (49.4)	

*hsTnI* high sensitive troponin-I

**Fig. 3:**
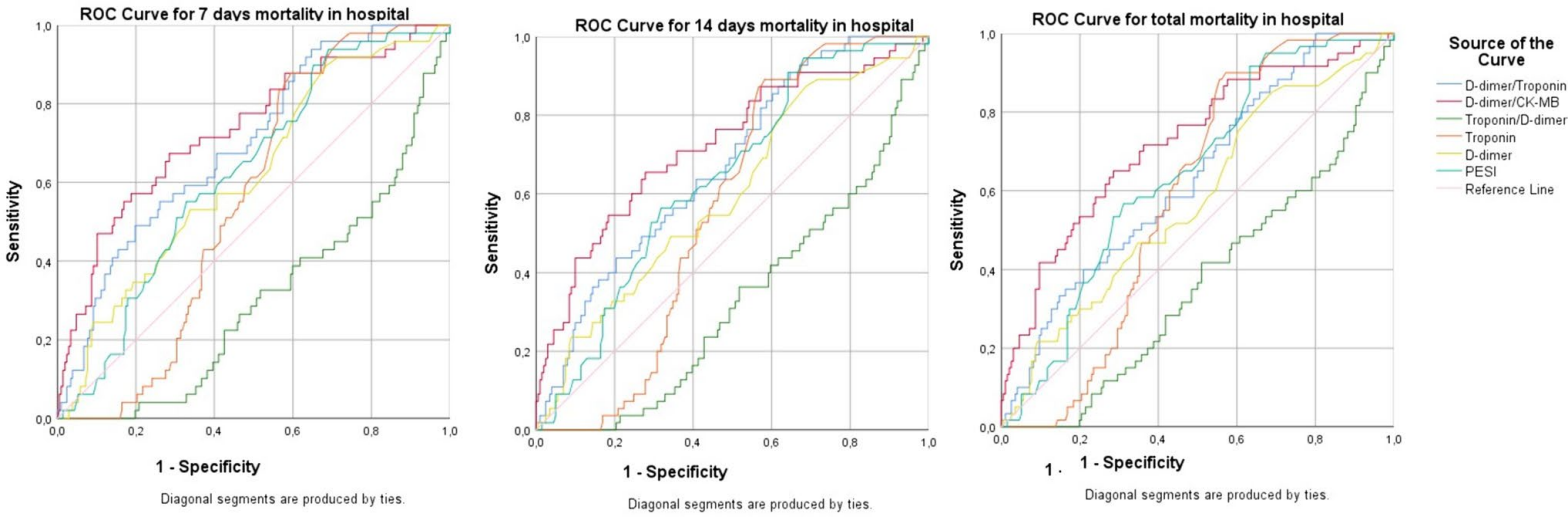
Looking at the success of the indices calculated from laboratory tests of the cases in predicting mortality

Kaplan–Meier survival analysis showed significant survival differences in in-hospital all-cause mortality at 30 days. The highest mortality risk was seen in Group 4, while the mortality risk was lower in Group 1 (Fig. 3).

The success of D-dimer/HsTroponin, D-dimer/CK-MB and troponin/D-dimer indices calculated from the laboratory test results of the cases in predicting mortality was examined, and a comparison was made with the success of the PESI score in predicting mortality. Among these indices, D-dimer/CK-MB was found to be the most successful index in predicting 7-day mortality (AUC: 0.734; 95% CI: 0.653–0.815; *p* < 0.001). Additionally, the D-dimer/HsTroponin ratio was found to be statistically significant as a successful index in predicting 7-day mortality (AUC: 0.697; 95% CI: 0.621–0.774; *p* < 0.001). It was observed that these two indices had a higher AUC level than PESI’s success in predicting 7-day mortality (AUC: 0.626; 95% CI: 0.547–0.704; *p* = 0.040) and had a statistical significance level (Table 3 and Fig. 3).

**Table 3:** Looking at the success of the indices calculated from the laboratory tests of the cases in predicting mortality

Parameter		Cutoff	AUC	Sensitivity	Specificity	*p*	%95 CI
In-hospital mortality (7 days)	D-dimer/hsTnI	96.39	0.697	93.9	35.3	** < 0.001**	0.621–0.774
	D-dimer/CK-MB	4541.33	0.734	67.3	71.5	** < 0.001**	0.653–0.815
	hsTnI	19.50	0.554	87.8	40.6	0.239	0.484–0.624
	D-dimer	3.15	0.619	53.1	66.2	**0.010**	0.535–0.703
	PESI	83.50	0.626	89.8	32.4	**0.040**	0.547–0.704
In-hospital mortality (14 days)	D-dimer/hsTnI	96.39	0.673	90.9	35.3	** < 0.001**	0.599–0.748
	D-dimer/CK-MB	2893.75	0.724	76.4	54.2	** < 0.001**	0.645–0.804
	hsTnI	19.50	0.564	89.1	41.8	0.147	0.496–0.632
	D-dimer	1.55	0.598	87.3	31.3	**0.026**	0.515–0.682
	PESI	86.5	0.639	90.9	35.8	**0.002**	0.563–0.00
In-hospital mortality (30 days)	D-dimer/hsTnI	27.01	0.635	100	19.9	**0.002**	0.559–0.712
	D-dimer/CK-MB	2026.15	0.724	88.3	42.3	** < 0.001**	0.679–0.800
	hsTnI	19.50	0.578	90.0	42.9	**0.035**	0.522–0.658
	D-dimer	1.55	0.578	85.0	33.11	**0.069**	0.496–0.659
	PESI	86.5	0.643	91.7	36.7	**0.001**	0.570–0.716

*hsTnI* high sensitive troponin-I, *CK-MB* creatine kinase-MB, *PESI* Pulmonary Embolism Severity Index

Values of *p*<0.05 are indicated in bold

Likewise, when looking at the success of these indexes in predicting 15-day and 30-day in-hospital mortality and comparing them with the predictive success of the PESI score, D-dimer/CK-MB was the most successful index in predicting both 15-day mortality and 30-day mortality (AUC: 0.724, 95% CI: 0.645-0.804, *p* < 0.001; AUC: 0.724, 95% CI: 0.679–0.800, *p* < 0.001) and was significantly higher than the success of the PESI score in predicting 15-day and 30-day mortality (Table 3 and Fig. 3).

## Discussion

Acute pulmonary embolism is a common, recurrent cardiovascular disease with high mortality and morbidity rates. Difficulties and delays in the diagnosis process of pulmonary embolism cause increased mortality. In addition, the scoring systems used to determine the prognosis of pulmonary embolism are difficult to use because they are time consuming. Therefore, there is a need for powerful, inexpensive and easily accessible markers that can be used in the diagnosis and prognosis of pulmonary embolism. Our study reports that D-dimer/High Sensitive Troponin ratio (D-dimer/HsTroponin) can be used as a mortality marker in patients with pulmonary embolism.

Our study was conducted on 256 pulmonary embolism cases, 46.5% of whom were male and the average age of the cases was 67.62 ± 15.42 years. In another study conducted on pulmonary embolism patients, the male gender ratio was 40%, while the median age was reported to be 68.5 years [11]. In another study, the average age of the patients was 58.2 ± 9.4 years, and 43.3% were found to be male [12]. The demographic characteristics of the case group of our study are similar to the literature. We think that the population of the disease is similar due to female gender and advanced age, which are among the risk factors of pulmonary embolism, and additional comorbidities and immobilization.

The 30-day mortality rate in the patients included in our study was determined to be 23.5%. In the study conducted by Kara et al., the mortality rate was found to be 31% [13]. Another study reported this rate to be 8.7% [14]. The mortality rate in our study is similar to the literature. Pulmonary embolism results in death, especially as a result of right ventricular dysfunction resulting from increased pressure distal to the thrombus. We think that the mortality rate is similar as the development of right ventricular dysfunction develops at different rates depending on the size and location of the thrombus. Many hematological, biochemical tests and scoring systems are used to determine the prognosis of pulmonary embolism patients. The most commonly used laboratory parameters are D-dimer and troponin tests. As a result of the study conducted by Kasapoğlu et al., it was reported that the D-dimer test showed a significant difference between the patients who survived and the patients who died [15]. Another study showed that the D-dimer value was significantly higher in the patients who died [16]. Similarly, in our study, D-dimer was found to be significantly higher in the group that resulted in exitus.

Although it was emphasized in a study that hsTnT could be used as an additional test to exercise testing and myocardial perfusion scintigraphy, there is no data indicating that hsTnT can be added to the diagnostic tools used in pulmonary embolism [17]. Again, it has also been reported that high sensitive troponin T (HsTn) is an indicator of 30-day mortality in pulmonary embolism [18]. According to the meta-analysis conducted by Becattini et al., the prognostic value of troponin was shown to be consistent among 20 studies [19]. In our study, it was found to be significant as a prognosis indicator, similar to the literature. The reason why these situations are similar is that as a result of the large size of the thrombus and the affected organs being in parallel, the level of D-dimer, which is a fibrin degradation product, increases and the troponin value increases with the development of right ventricular dysfunction. In our study, we think that since HsTn was used in particular, it could predict mortality at a higher rate if it increased at an earlier period.

Pulmonary Embolism Severity Index (PESI), which is used as a prognostic indicator of pulmonary embolism, is the most commonly used indicator. PESI has been found to be effective in predicting long-term mortality, especially as a 30-day mortality marker [20]. Studies have shown that the PESI score has a higher predictive value in terms of patient mortality than other scores [21]. Another study found that having a higher PESI score in the group of deceased patients showed a higher prognostic value [22]. In our study, it was found that there was a significant difference in PESI score between the deceased patient group and the living patient group. PESI score can detect patients with multiorgan involvement at a higher rate due to the inclusion of many parameters. Therefore, those with higher scores have a higher risk. But in general, its use is time-consuming and not highly applicable in the emergency department setting.

Our study shows that the high D-dimer/HsTroponin ratio can be used instead of the PESI score as a marker of 7, 14 and 30-day in-hospital mortality. In particular, while the D-dimer/HsTroponin ratio cut-off value was 96.39, it was observed to have higher sensitivity and specificity than the PESI score, with a sensitivity of 93.9% and a specificity of 35.3%. When the literature was scanned, no previous study was found on the mortality of the D-dimer/HsTroponin ratio in pulmonary embolism patients. However, in a study where the D-dimer/TnI ratio was used in the differential diagnosis of pulmonary embolism and NonSTEMI, it was found to be significant in diagnosing pulmonary embolism [10]. It is widely used especially in acute coronary syndrome related to HsTnT, and there are very few studies showing pulmonary embolism mortality [7]. In studies conducted with HsTnT, it has been reported to be significant as a mortality marker in patients with cardiovascular disease and a history of major cardiovascular surgery [23, 24].

In particular, our study is a study conducted with HsTn and is the first to compare it with the PESI score. Our study found that the D-dimer/HsTroponin ratio, as a cheap, easy and powerful test during the diagnosis of patients with pulmonary embolism, is more meaningful than the PESI score as an indicator of mortality.

## Limitations

The most important limitation of this study is that it was designed as a retrospective study. Other limitations include the single-center nature of the study and the relatively small number of cases. Another limitation is that it determines the short-term mortality outcomes of the patients included in the study. Prospective studies with a higher number of cases are needed to confirm the predictive values ​​of the parameters investigated in this study.

## Conclusion

We think that the D-dimer/HsTroponin ratio, which is a powerful, fast, low-cost, easy and simple test, can be used especially in emergency services instead of the PESI score as a mortality marker in pulmonary embolism, which has a high mortality rate.

## Data Availability

Data and materials are reachable from hospital automation information systems.
